# Relationship Between Acylcarnitine and the Risk of Retinopathy in Type 2 Diabetes Mellitus

**DOI:** 10.3389/fendo.2022.834205

**Published:** 2022-03-15

**Authors:** Wan-Ying Wang, Xu Liu, Xiao-Qian Gao, Xin Li, Zhong-Ze Fang

**Affiliations:** Department of Toxicology and Sanitary Chemistry, School of Public Health, Tianjin Medical University, Tianjin, China

**Keywords:** acylcarnitine, diabetic retinopathy, type 2 diabetes mellitus, metabonomics, diabetic complications

## Abstract

**Objective:**

Diabetic retinopathy is a common complication of type 2 diabetes mellitus (T2DM). Due to the limited effectiveness of current prevention and treatment methods, new biomarkers are urgently needed for the prevention and diagnosis of DR. This study aimed to explore the relationships between plasma acylcarnitine with DR in T2DM.

**Methods:**

From May 2015 to August 2016, data of 1032 T2DM patients were extracted from tertiary hospitals. Potential non-linear associations were tested by binary logistic regression models, and ORs and 95% CIs of the research variables were obtained. Correlation heat map was used to analyze the correlation between variables. The change of predictive ability was judged by the area under the receiver operating characteristic curve.

**Results:**

Of the 1032 patients with T2DM, 162 suffered from DR. After adjusting for several confounding variables, C2 (OR:0.55, 95%CI:0.39-0.76), C14DC (OR:0.64, 95%CI:0.49-0.84), C16 (OR:0.64, 95%CI:0.49-0.84), C18:1OH (OR:0.51, 95%CI:0.36-0.71) and C18:1 (OR:0.60, 95%CI:0.44-0.83) were negatively correlated with DR. The area under the curve increased from 0.794 (95% CI 0.745 to 0.842) to 0.840 (95% CI 0.797 to 0.833) when C2, C14DC, C18:1OH and C18:1 added to the traditional risk factor model.

**Conclusion:**

There was a negative correlation between C2, C14DC, C16, C18:1OH, and C18:1 and the risk of retinopathy in patients with T2DM. C2, C14DC, C18:1OH, and C18:1 may be new predictors and diagnostic markers of DR.

## Introduction

Diabetes is a chronic disease prevalent all over the world. It is estimated that the global prevalence of diabetes was about 9.3% in 2019, and it will rise to 10.2% by 2030 and 10.9% by 2045 (1). China currently has the largest number of diabetic patients in the world (2). Diabetic retinopathy(DR), as a common microvascular complication of type 2 diabetes mellitus (T2DM), is the main cause of visual impairment and blindness among working-age people, which brings a heavy burden to society and families (3). The main treatments of DR include anti-vascular endothelial growth factor therapy, laser photocoagulation, corticosteroids, and vitrectomy (4). Since these invasive treatments are mainly used in the late stage of irreversible injury, we need new biomarkers for the prevention and screening of DR (5).

The pathogenesis of DR is complex, involving a variety of vascular, inflammatory, and neural mechanisms (6–8). Inflammation mediates structural and molecular alterations related to DR. However, the molecular mechanism of DR-related inflammatory pathways has not been fully elucidated (9). Studies have shown that different fatty acid (FA) metabolism will produce different inflammatory effects (10–13). As an intermediate of FA metabolism, the level of acylcarnitine (AC) is closely related to FA metabolism (14–16). The level of ACs reflects FAs metabolism in obese and T2DM people (17). As a marker of inflammation, the level of ACs was different from that of healthy and lean humans (18, 19). In this study, we aimed to test the associations between plasma AC levels and risk of DR. Determining the difference in AC profile is helpful to find new targets for early diagnosis of DR.

## Materials and Methods

### Research Design and Study Patients

In this study, a cross-sectional design was used to investigate the relationship between AC and DR. The objects and methods of this study have been described in detail in previous studies (20). In short, from May 2015 to August 2016, the Liaoning Medical University First Affiliated Hospital (LMUFAH) had 1,898 consecutive inpatients with T2DM. T2DM meets the diagnostic criteria of the WHO in 1999 (21). Their electronic medical records were retrieved and metabolomics profiles were measured. 1032 patients older than 18 years with complete information of anthropometric and AC metabolism and a clear DR diagnosis were eventually enrolled. The LMUFAH Clinical Research Ethics Committee approved the ethics of this study. Due to the retrospective nature of the study, informed consent was exempted, consistent with the Declaration of Helsinki.

### Data Collection and Definitions

Information of demographic and anthropometric, diabetes duration, clinical and laboratory measurements, and diagnosis of DR was recorded in the electronic medical records. Clinical indicators include systolic blood pressure (SBP), diastolic blood pressure (DBP), glycosylated hemoglobin (HbA1c), triglycerides (TG), high-density lipoprotein cholesterol (HDL-C), and low-density lipoprotein cholesterol (LDL-C).

### Clinical Definitions

DR was defined as the presence of microaneurysm, soft exudate, hard exudate, retinal hemorrhage, or vitreous hemorrhage, which was evaluated by binocular retinography (22). The SBP and DBP of the patients were measured by a calibrated mercury sphygmomanometer after sitting for 5 to 10 minutes. Body mass index (BMI) was calculated by dividing weight in kilograms by height in meters squared. According to the WHO’s Asian body mass index standards, BMI was divided into four categories: underweight (<18.5 kg/m^2^), normal weight (18.5-23.9 kg/m^2^), overweight (24.0-27.9 kg/m^2^), and obesity (≥28.0 kg/m^2^) (23). Hyperglycemia and dyslipidemia were defined as failure to meet the treatment goals recommended by the American Diabetes Association, that is, HbA1c≥7% as hyperglycemia; TG≥1.7 mmol/L, LDL-C≥2.6 mmol/L, male HDL-C ≤ 1 mmol/L or female HDL-C ≤ 1.3 mmol/L as dyslipidemia (24).

AC was divided into short-chain AC (C2 to C6), medium-chain AC (C8 to C14), and long-chain AC (C16 to C26) according to the number of carbon atoms in the acyl chain (25).

### Acylcarnitine Quantification

Methods of AC had been described in previous studies (26). Briefly, the dry blood spot samples of finger puncture were collected after fasting for 8 hours, and the samples were detected by mass spectrometry (MS) technology. The MS metabolic analysis was performed using the AB Sciex 4000 QTrap system (AB Sciex, Framingham, MA, USA). The electrospray ionization source was an ion source. The ion spray voltage was 4.5kV. Positive mode was performed to scan analytes. Each dried sample was dissolved in 100μL fresh mobile phase solution. The mobile phase was 80% acetonitrile aqueous solution. Absolute quantification used AC isotope-labeled internal standard from Cambridge Isotope Laboratories (Tewksbury, Massachusetts, USA). Mixed and dissolved the standards in 2mL pure methanol and stored it at 4°C. Working solution was prepared through 100-fold dilution for metabolite extraction.

### Statistical Analysis

Continuous variables with a normal distribution are reported as mean (SD), and skewed variables are reported as median (IQR). Check normality by observing the Q-Q plot. Categorical variables are expressed in frequency (%). Participants were divided into two groups according to their DR status. The non-paired Student t-test for normally distributed variables or the Mann-Whitney U test for skewed distribution variables were used to compare the differences between the two groups. The χ^2^ test or the eligible Fisher test was used to compare the differences between the two groups of categorical variables.

A single-factor binary logistic regression model was used to obtain the OR value and 95% confidence interval of the degree of the reaction AC on the DR. Use structural adjustment programs to control the confounding effects of other variables. First, obtain an unadjusted OR; secondly, perform multivariate analysis to adjust traditional risk factors, including age, gender, SBP, DBP, BMI, and diabetes duration (multivariate model 1); finally, add HDL-C, LDL-C, HbA1c, and TG (multivariate model 2) based on model 1, to obtain the adjusted OR value of AC with statistical significance.

The correlation heat map was drawn to analyze the correlation of the incoming variables adjusted by logistic regression. Delete the related variables with less contribution to the model. The correlation coefficient is r ≥ 0.8, indicating a significant correlation between variables. Receiver operating characteristic curve (ROC) was drawn to compare the difference of area under the ROC curve (AUC) and determine whether the prediction ability of the DR model increased after adding AC.

P values of <0.05 were considered statistically significant and were corrected by Bonferroni. All analyses were performed using R V.4.0. R packets used include caret, randomForest, tableone survival, epiDisplay, and kernlab.

## Result

### Characteristics of the Study Patients

The average age of 1032 T2DM patients included in this study was 57.2 (SD 13.8) years, of which 53.2% were males. The average BMI of the study population was 25.3 (SD 3.9) kg/m^2^, of which overweight and obesity accounted for 63.1%. There were 162 patients with DR. The median diabetes duration in the DR group was 13 (IQR 6-20) years, significantly higher than that in the control group (M 4, IQR 0-10). In addition, compared with the non-DR group, there were more women in the DR group, higher SBP, lower HbA1c levels, and a higher proportion of patients with abnormal levels of TG and LDL-C. Other characteristics were similar between the two groups (**Table 1**).

**Table 1:** Characteristics of patients with T2DM according to DR status.

Variables	Total^a^ (n=1032)	DR^b^ (n=162)	Non-DR^c^ (n=870)	P Value^d^
Age, mean (SD), years	57.2 (13.8)	57.8 (10.0)	57.1 (14.4)	.591
Sex, male, No. (%)	549 (53.2)	73 (45.1)	476 (54.7)	.03
Diabetes duration, median (IQR), years	5 (0 - 10)	13 (6 - 20)	4 (0 - 10)	<.001
Body mass index, mean (SD), kg/m^2^	25.3 (3.9)	25.1 (3.3)	25.3 (3.9)	.371
~18.5, No. (%)^e^	27 (2.6)	1 (0.6)	26 (3.0)	.165
18.5~24.0, No. (%)^e^	354 (34.3)	64 (39.5)	288 (33.1)	
24.0~28.0, No. (%)^e^	430 (41.7)	66 (40.7)	366 (42.1)	
28.0~, No. (%)^e^	221 (21.4)	31 (19.1)	190 (21.8)	
SBP, mean (SD), mm Hg		145.6 (25.3)	139.5 (23.6)	.003
DBP, mean (SD), mm Hg		83.0 (13.4)	82.4 (13.5)	.551
HbA1C, median (IQR), %		9.56 (8.76 - 10.09)	9.80 (8.70 - 10.20)	.024
<7, No. (%)		15 (9.3)	62 (7.1)	.432
≥7, No. (%)		147 (90.7)	808 (92.9)	
Triglyceride, median (IQR), mmol/L		1.96 (1.59 - 2.17)	1.98 (1.29 - 2.20)	.517
<1.70, No. (%)		46 (28.4)	337 (38.7)	.016
≥1.70, No. (%)		116 (71.6)	533 (61.3)	
HDL-C, median (IQR), mmol/L		1.08 (1.00 - 1.13)	1.08 (0.89 - 1.14)	.198
>1.00 in men or >1.30 in women, No. (%)		68 (42.0)	337 (38.7)	.492
≤1.00 in men or ≤1.30 in women, No. (%)		94 (58.0)	533 (61.3)	
LDL-C, median (IQR), mmol/L		2.91 (2.66 - 3.02)	2.93 (2.40 - 3.13)	.698
<2.60, No. (%)		38 (23.5)	269 (30.9)	.07
≥2.60, No. (%)		124 (76.5)	601 (69.1)	

T2DM, type 2 diabetes; DR, diabetic retinopathy; SD, standard deviation; IQR, interquartile range; SBP, systolic blood pressure; DBP, diastolic blood pressure; HbA1c, glycated hemoglobin; HDL-C, high-density lipoprotein cholesterol; LDL-C, low-density lipoprotein cholesterol.

a All subjects were analyzed only for age, sex, diabetes duration, body mass index, and body mass index categories.

b Type 2 diabetic patients with retinopathy.

c Patients with type 2 diabetes mellitus without retinopathy.

d P value was acquired by comparing DR and non-DR and was derived from independent-samples Student t-test for normally distributed variables, Mann-Whitney U test for skewed distributions, and χ^2^ test or the eligible Fisher test for categorical variables.

e Fisher’s exact test was used.

The results showed that the levels of C2, C3, C4, C5, C6DC, C14, C14DC, C14:1, C16, C16OH, C16:1OH, C18, C18OH, C18:1, C18:1OH, C20, C22, and C26 in the DR group were lower than those in the control group, and the contents of other AC were similar in the two groups (**Table 2**).

**Table 2:** Acylcarnitine profile in T2DM patients.

Variables	DR^a^	Non-DR^b^	P Value^c^
C2, median (IQR), μmol/L	10.44 (8.17 to 12.90)	11.97 (8.99 to 15.85)	<.001
C3, median (IQR), μmol/L	1.18 (0.84 to 1.49)	1.41 (0.97 to 2.00)	<.001
C3DC, median (IQR), μmol/L	0.09 (0.06 to 0.13)	0.09 (0.07 to 0.12)	.754
C4, median (IQR), μmol/L	0.19 (0.15 to 0.25)	0.21 (0.15 to 0.28)	.036
C4DC, median (IQR), μmol/L	0.63 (0.47 to 0.81)	0.65 (0.49 to 0.86)	.381
C5, median (IQR), μmol/L	0.12 (0.09 to 0.17)	0.15 (0.11 to 0.20)	<.001
C5OH, median (IQR), μmol/L	0.24 (0.19 to 0.34)	0.27 (0.20 to 0.36)	.076
C5DC, median (IQR), μmol/L	0.07 (0.05 to 0.12)	0.08 (0.05 to 0.12)	.145
C5:1, median (IQR), μmol/L	0.06 (0.04 to 0.08)	0.06 (0.05 to 0.08)	.215
C6, median (IQR), μmol/L	0.05 (0.03 to 0.06)	0.05 (0.04 to 0.07)	.051
C6DC, median (IQR), μmol/L	0.75 (0.51 to 0.98)	0.81 (0.62 to 1.08)	.009
C8, median (IQR), μmol/L	0.06 (0.04 to 0.10)	0.07 (0.05 to 0.10)	.691
C10, median (IQR), μmol/L	0.09 (0.06 to 0.16)	0.09 (0.06 to 0.15)	.668
C10:1, median (IQR), μmol/L	0.11 (0.07 to 0.19)	0.11 (0.08 to 0.16)	.93
C10:2, median (IQR), μmol/L	0.73 (0.49 to 1.11)	0.77 (0.54 to 1.06)	.723
C12, median (IQR), μmol/L	0.05 (0.04 to 0.07)	0.05 (0.04 to 0.07)	.247
C14, median (IQR), μmol/L	0.06 (0.05 to 0.08)	0.07 (0.05 to 0.09)	.001
C14OH, median (IQR), μmol/L	0.05 (0.04 to 0.06)	0.05 (0.04 to 0.07)	.121
C14DC, median (IQR), μmol/L	0.04 (0.02 to 0.05)	0.04 (0.03 to 0.06)	<.001
C14:1, median (IQR), μmol/L	0.09 (0.06 to 0.12)	0.10 (0.07 to 0.13)	.011
C14:2, median (IQR), μmol/L	0.71 (0.45 to 0.91)	0.71 (0.51 to 0.99)	.245
C16, median (IQR), μmol/L	0.80 (0.65 to 0.96)	0.94 (0.74 to 1.20)	<.001
C16OH, median (IQR), μmol/L	0.02 (0.02 to 0.03)	0.03 (0.02 to 0.04)	.036
C16:1OH, median (IQR), μmol/L	0.05 (0.04 to 0.06)	0.06 (0.04 to 0.07)	<.001
C18, median (IQR), μmol/L	0.41 (0.33 to 0.50)	0.46 (0.36 to 0.58)	.001
C18OH, median (IQR), μmol/L	0.02 (0.02 to 0.03)	0.03 (0.02 to 0.03)	.013
C18:1, median (IQR), μmol/L	0.57 (0.48 to 0.68)	0.65 (0.52 to 0.84)	<.001
C18:1OH, median (IQR), μmol/L	0.03 (0.02 to 0.03)	0.03 (0.02 to 0.05)	<.001
C18:2, median (IQR), μmol/L	1.66 (1.27 to 2.06)	1.73 (1.28 to 2.18)	.26
C20, median (IQR), μmol/L	0.04 (0.04 to 0.05)	0.05 (0.04 to 0.06)	.028
C22, median (IQR), μmol/L	0.07 (0.05 to 0.09)	0.08 (0.06 to 0.10)	.001
C24, median (IQR), μmol/L	0.05 (0.04 to 0.07)	0.06 (0.04 to 0.07)	.192
C26, median (IQR), μmol/L	0.03 (0.02 to 0.05)	0.04 (0.03 to 0.05)	.029

T2DM, type 2 diabetes mellitus; DR, diabetic retinopathy; IQR, interquartile range; C2, acetylcarnitine; C3, propionylcarnitine; C3DC, propionylcarnitine; C4, butyrylcarnitine; C4DC, succinylcarnitine; C5, isovalerylcarnitine; C5OH, 3-hydroxyisovalerylcarnitine; C5DC, glutarylcarnitine; C5:1, tiglylcarnitine; C6, hexanoylcarnitine; C6DC, adipylcarnitine; C8, octanoylcarnitine; C10, decanoylcarnitine; C10:1, decanoylcarnitine; C10:2, sebacoylcarnitine; C12, lauroylcarnitine; C14, myristoylcarnitine; C14OH, 3-hydroxyl-tetradecanoylcarnitine; C14DC, tetradecanoyldiacylcarnitine; C14:1, tetradecenoylcarnitine; C14:2, tetradecadienylcarnitine; C16, palmitoylcarnitine; C16OH, 3-hydroxypalmitoylcarnitine; C16:1OH, 3-hydroxypalmitoleylcarnitine; C18, octadecanoylcarnitine; C18OH, 3-hydroxy-octadecoylcarnitine; C18:1, octacarbonylcarnitine; C18:1OH, 3-hydroxy-octadecylcarnitine; C18:2, octadecadienylcarnitine; C20, arachidic carnitine; C22, behenic carnitine; C24, tetracosanoic carnitine; C26, hexacosanoic carnitine.

a Type 2 diabetic patients with retinopathy.

b Patients with type 2 diabetes mellitus without retinopathy.

c P value was acquired by comparing DR and non-DR and was derived from the Mann-Whitney U test for skewed distributions.

### Associations of Acylcarnitines With DR Risk

**Table 3** shows the results of univariate logistic regression. C2, C3, C5, C6, C14:1, C14DC, C16, C16:1OH, C18, C18:1, C18:1OH, C18OH, C20, and C22 were adjusted for p<0.05, OR < 1, which was negatively correlated with risk of DR. Add traditional risk factors (age, gender, BMI, SBP, BP, and diabetes duration) to the univariate logistic regression model to adjust for confounding factors (multivariate model 1) and get C2, C14DC, C16, C18:1, and C18:1OH P-value <0.05. Based on multivariate model 1, we continue to adjust HbA1c, TG, HDL-C, and LDL-C (multivariate model 2). The effects of C2, C14DC, C16, C18:1OH, and C18 were almost unchanged (**Table 4**). See **Supplementary Material** for all results of multivariate analysis.

**Table 3:** OR of acylcarnitine for DR risk in T2DM (univariable model)^a^.

Acylcarnitine	OR	95%CI	P Value^b^
C2	0.59	0.45 to 0.77	.001
C3	0.61	0.47 to 0.78	<.000
C3DC	0.9937	0.80 to 1.24	.955
C4	0.78	0.62 to 0.98	.077
C4DC	0.89	0.71 to 1.11	.386
C5	0.62	0.49 to 0.80	.001
C5:1	0.92	0.74 to 1.14	.505
C5DC	0.78	0.57 to 1.09	.216
C5OH	0.95	0.76 to 1.18	.694
C6	0.72	0.56 to 0.91	.021
C6DC	0.58	0.29 to 1.14	.184
C8	0.88	0.71 to 1.10	.369
C10	0.89	0.71 to 1.11	.386
C10:1	0.89	0.71 to 1.10	.370
C10:2	0.93	0.74 to 1.16	.551
C12	0.87	0.70 to 1.09	.323
C14	0.79	0.62 to 0.99	.078
C14:1	0.69	0.53 to 0.89	.016
C14:2	0.81	0.65 to 1.02	.134
C14DC	0.62	0.49 to 0.79	.001
C14OH	0.8	0.62 to 1.03	.139
C16	0.58	0.45 to 0.75	<.000
C16:1OH	0.71	0.57 to 0.90	.016
C16OH	0.81	0.65 to 1.02	.138
C18	0.72	0.57 to 0.90	.017
C18:1	0.54	0.41 to 0.71	<.000
C18:1OH	0.51	0.38 to 0.69	<.000
C18:2	0.83	0.67 to 1.04	.175
C18OH	0.76	0.60 to 0.95	.043
C20	0.69	0.53 to 0.90	.021
C22	0.76	0.61 to 0.95	.043
C24	0.97	0.78 to 1.21	.845
C26	0.91	0.73 to 1.13	.471

T2DM, type 2 diabetes mellitus; DR, diabetic retinopathy; OR, odds ratio; CI, confidence interval; C2, acetylcarnitine; C3, propionylcarnitine; C3DC, propionylcarnitine; C4, butyrylcarnitine; C4DC, succinylcarnitine; C5, isovalerylcarnitine; C5OH, 3-hydroxyisovalerylcarnitine; C5DC, glutarylcarnitine; C5:1, tiglylcarnitine; C6, hexanoylcarnitine; C6DC, adipylcarnitine; C8, octanoylcarnitine; C10, decanoylcarnitine; C10:1, decanoylcarnitine; C10:2, sebacoylcarnitine; C12, lauroylcarnitine; C14, myristoylcarnitine; C14OH, 3-hydroxyl-tetradecanoylcarnitine; C14DC, tetradecanoyldiacylcarnitine; C14:1, tetradecenoylcarnitine; C14:2, tetradecadienylcarnitine; C16, palmitoylcarnitine; C16OH, 3-hydroxypalmitoylcarnitine; C16:1OH, 3-hydroxypalmitoleylcarnitine; C18, octadecanoylcarnitine; C18OH, 3-hydroxy-octadecoylcarnitine; C18:1, octacarbonylcarnitine; C18:1OH, 3-hydroxy-octadecylcarnitine; C18:2, octadecadienylcarnitine; C20, arachidic carnitine; C22, behenic carnitine; C24, tetracosanoic carnitine; C26, hexacosanoic carnitine.

a Univariate logistic regression analysis of 33 kinds of acylcarnitine and DR were done respectively.

b P value corrected by Bonferroni.

**Table 4:** OR of Acylcarnitine for DR risk in T2DM (Multivariable model)^a^.

	OR	95%CI	P Value^b^
**Multivariable Model 1** ^**c**^			
C18:1OH	0.50	0.36 to 0.69	.001
C2	0.58	0.43 to 0.80	.011
C16	0.64	0.49 to 0.85	.016
C14DC	0.66	0.51 to 0.86	.016
C18:1	0.63	0.46 to 0.85	.016
**Multivariable Model 2** ^**d**^			
C18:1OH	0.51	0.36 to 0.71	.003
C2	0.55	0.39 to 0.76	.006
C14DC	0.64	0.49 to 0.84	.010
C16	0.63	0.47 to 0.83	.010
C18:1	0.60	0.44 to 0.83	.012

T2DM, type 2 diabetes mellitus; DR, diabetic retinopathy; OR, odds ratio; CI, confidence interval; C18:1OH, 3-hydroxy-octadecylcarnitine; C2, acetylcarnitine; C16, palmitoylcarnitine; C14DC, tetradecanoyldiacylcarnitine; C18:1, octacarbonylcarnitine.

a Only acylcarnitines with significant results were listed.

b P value corrected by Bonferroni.

c Multivariable model 1 was adjusted for age, sex, body mass index (<18.5 kg/m2, 18.5 kg/m2-24.0 kg/m2, 24.0 kg/m2-28.0 kg/m2 and >28.0 kg/m2), SBP, DBP, and diabetes duration on the basis of univariable model.

d Multivariable model 2 was adjusted for variables in multivariable model 1 plus glycated hemoglobin (<7%, ≥7%), triglyceride (<1.7mmol/L, ≥1.7mmol/L), high-density lipoprotein cholesterol (<1.0mmol/L in male or <1.3mmol/L in female, ≥1.0mmol/L in male or ≥1.3mmol/L in female), and low-density lipoprotein cholesterol (<2.6mmol/L, ≥2.6 mmol/L).

### Correlation Analysis Between Predictors

**Figure 1:**
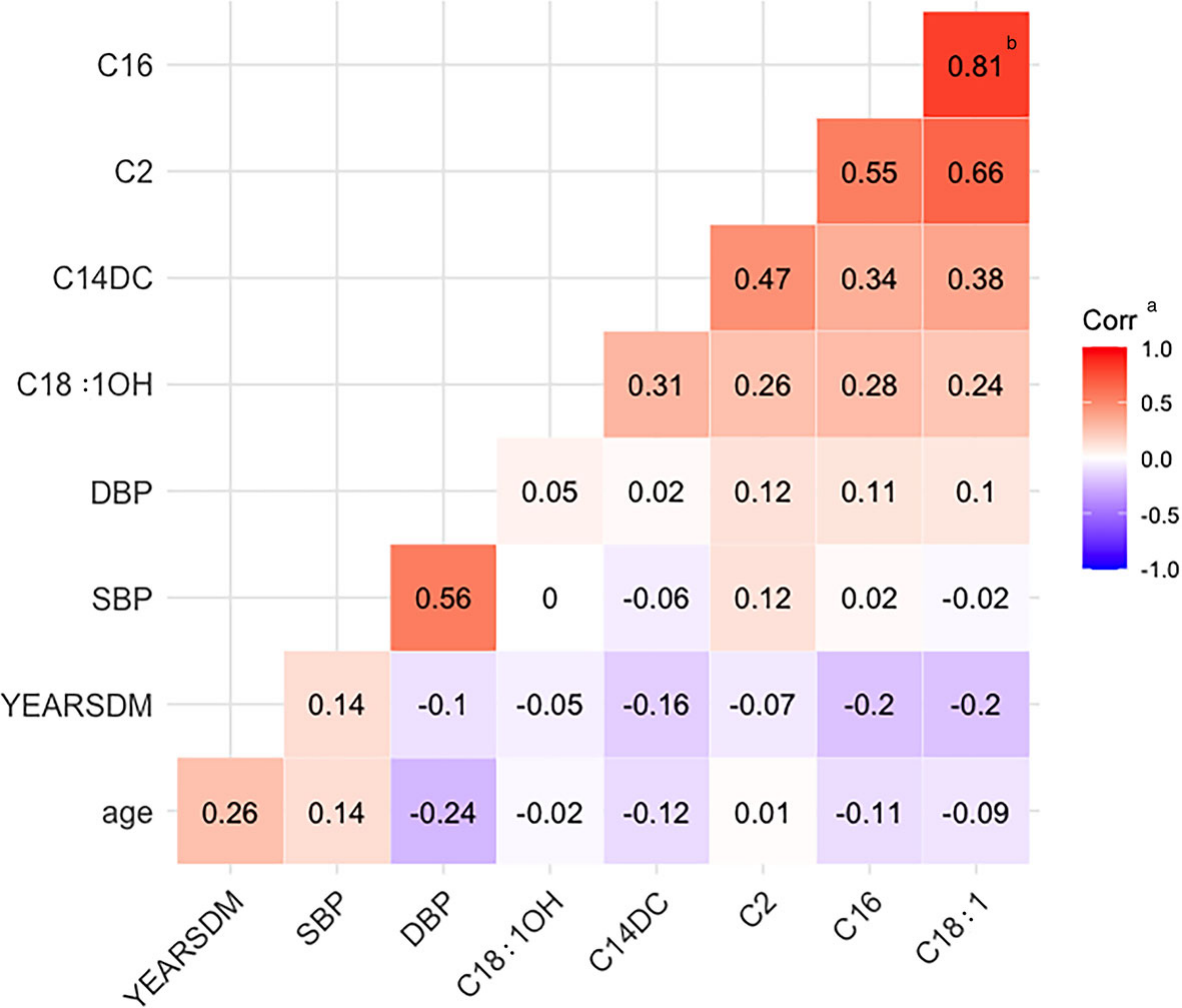
Heat map of correlation. YEARSDM, diabetes duration; SBP, systolic blood pressure; DBP, diastolic blood pressure; C18:1OH, 3-hydroxy-octadecylcarnitine; C14DC, tetradecanoyldiacylcarnitine; C2, acetylcarnitine; C16, palmitoylcarnitine; C18:1, octacarbonylcarnitine. ^a^Corr, the correlation coefficient r, red indicates positive correlation, blue indicates negative correlation, r > 0.8 indicates that the two variables correlate. ^b^The two positively correlated variables, C16 and C18:1, had a correlation coefficient of 0.81.

Correlation analysis of age, SBP, DBP, diabetes duration, C2, C14DC, C16, C18:1, and C18:1OH showed that the correlation coefficient between C16 and C18:1 was 0.81 (p<0.05) (**Figure 1**).

### Potential Increase in Predictive Values of Acylcarnitine for DR

**Figure 2:**
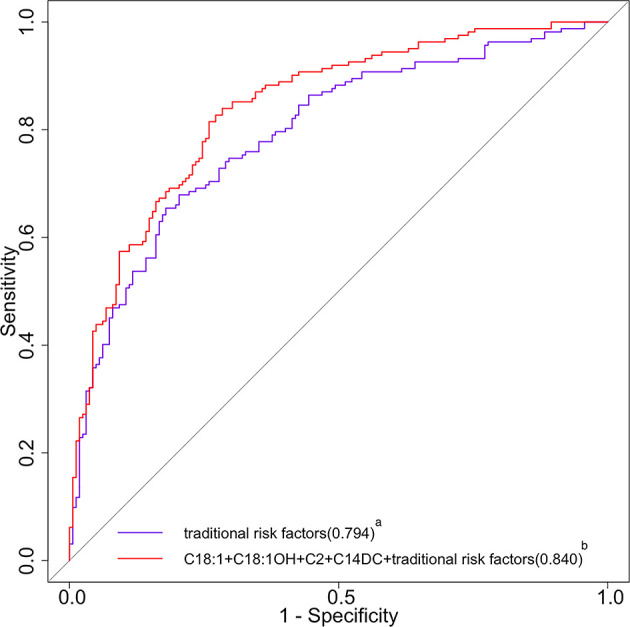
ROC curves of traditional risk factors and traditional risk factors plus acylcarnitine for DR. ROC, receiver operating characteristic; DR, diabetic retinopathy; C18:1, octacarbonylcarnitine; C18:1OH, 3-hydroxy-octadecylcarnitine; C2, acetylcarnitine; C14DC, tetradecanoyldiacylcarnitine. ^a^The purple curve stands for the traditional risk factor model (multivariable model 1 in **Table 4** for the list of variables); the area under the operating characteristic curve was 0.794 (95% CI 0.745 to 0.842) for the traditional risk factor model. ^b^The red curve stands for the traditional risk factors plus C18, C18:1OH, C2, and C14DC; the area under the operating characteristic curve was 0.840 (95% CI 0.797 to 0.883) for the traditional risk factor plus C18, C18:1OH, C2, and C14DC (p<0.05 for comparison of the traditional risk factor model).

The AUC was significantly increased by adding C2, C14DC, C18:1OH, and C18:1 to the traditional risk factor model, and the OR value increased from 0.794 (95%CI, 0.745-0.842) to 0.840 (95%CI, 0.797-0.883) (p = 0.0018) (**Figure 2**). Since C16 contributed less variance to the logistic regression model, it was deleted.

## Discussion

In this cross-sectional study, we analyzed the effect of AC levels on the risk of DR in T2DM patients. It could be seen that the levels of C2, C14DC, C16, C18:1OH, and C18:1 were decreased in DR patients, which was significantly related to the reduced risk of DR. This relationship was independent of other AC and traditional diabetes risk factors, suggesting that the risk of DR in T2DM patients might increase with the decrease of these AC levels. The AUC of the model was improved by adding C2, C14DC, C18:1OH, and C18:1 to the traditional prediction model. These AC metabolites were mainly long-chain AC.

DR is an inflammatory disease characterized by the destruction of the blood-retinal barrier, inflammatory process, and increased vascular permeability (27). Chronic hyperglycemia can induce the occurrence of DR, because the blood glucose control of diabetic patients can not completely stop the progress of DR after the normal level, indicating that other factors are affecting the progress of DR besides hyperglycemia (28). At present, many studies have focused on the effect of lipid metabolism on metabolic disorders, but it is not clear how AC, an intermediate product of lipid metabolism, affects the occurrence and development of diseases.

**Figure 3:**
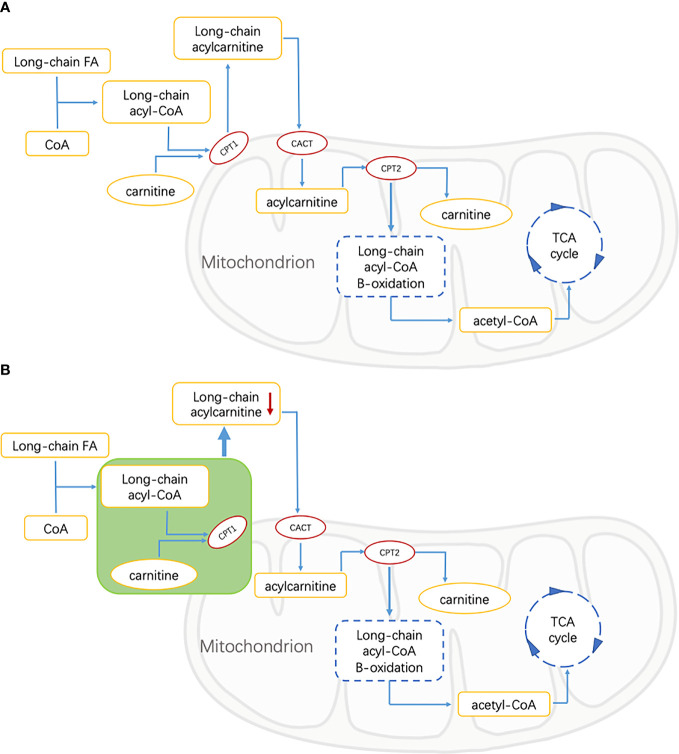
FA, fatty acid; CoA, Coenzyme A; CPT1, carnitine palmitoyltransferase 1; CACT, carnitine acylcarnitine translocase; CPT2, carnitine palmitoyltransferase 2; TCA, tricarboxylic acid. **(A)** Energy supply pathways of long-chain fatty acids in healthy people. Long-chain fatty acids are catalyzed by CPT1, CPT2, and CACT to produce acetyl-CoA, which is involved in the tricarboxylic acid cycle, and acylcarnitine is the intermediate metabolite. **(B)** Energy supply pathway of long-chain fatty acids in patients with diabetic retinopathy. The green part shows the inhibition of free FAs transport of CPT1-mediated that resulted in the decrease of long-chain acylcarnitine.

The lipid is an important part of the retina, which plays an important role in the function of the retina. Abnormal lipid metabolism is one of the important factors leading to the progress of DR (29, 30). In the process of mitochondrial metabolism of long-chain fatty acids (LCFAs), it is necessary to esterify carnitine with carnitine to form AC, and then proceed to the next step of metabolism (31). This is because neither LCFAs nor their coenzyme A (CoA) esters can pass through the mitochondrial inner membrane alone, so carnitine is an essential substance for LCFAs to enter the mitochondria through AC intermediates before β-oxidation (25). The acyl part can be transferred between CoA and carnitine through the reversible reaction of carnitine palmitoyltransferase I (CPT1) and II (CPT2). Carnitine binds the acyl residues on CoA to form free radicals and long-chain acyl-COAs (32). Long-chain acyl-COAs are converted to AC under the action of CPT1. Carnitine translocation enzyme (CACT) catalyzes AC to pass through the inner membrane of mitochondria (33). Then, the LCFAs are converted to long-chain acyl-CoA in the presence of CPT2 for β-oxidation. In this process, the number of carbon atoms of acyl-CoA decreases continuously, then enters the oxidation pathway of short-chain fatty acids (SCFAs), and finally produces acetyl-CoA to provide energy by participating in the tricarboxylic acid cycle (TCA cycle) (**Figure 3A**) (34).

As an important intermediate in the oxidation of fatty acids, the abnormal level of AC may be related to a disrupted FA metabolism in T2DM patients. Bene et al.’s studies on AC profile in patients with metabolic diseases showed that the levels of C3 and C4 in patients with T2DM were significantly higher than those in the control group, while the levels of some medium- and long-chain ACs were lower than those in controls. It was speculated that the decrease of these medium- and long-chain AC levels was affected by the inhibition of free FAs transport of CPT1-mediated (31, 35). This suggested that in our study, the decrease of long-chain ACs in the DR group may be related to the inhibition of long-chain FAs transport mediated by CPT1 (**Figure 3B**).

In the study of AC and insulin resistance, it is found that the incomplete β-oxidation of muscle FA led to the accumulation of AC, which in turn led to muscle oxidative stress and insulin resistance (36). The accumulation of long-chain AC in serum, muscle, and adipose tissue of insulin-resistant rats was observed in animal experiments (19). When the level of long-chain AC decreased, insulin sensitivity was effectively improved (37). However, Schooneman et al. believe that AC may only reflect the change of FA oxidation and has no correlation with insulin resistance (38). Combined with these studies, the association between ACs levels and insulin resistance remains unclear.

At present, there are few studies on the effects of AC metabolic disorders on DR. Some inconsistent results of diabetes and diabetes complications studies may be caused by differences in the study population and sample size, as well as study design and clinical phenotyping. In Fort PE et al.’s study, AC was detected in human retinal tissue and plasma samples. It was found that the abundance of ACs with carbon atom number ≥ 14 decreased in a gradient with the disease state of the subjects (without diabetes, diabetes without DR, and DR) (39). This finding suggested impaired mitochondrial β-oxidation of retinal FAs in the retina in DR which was consistent with our research. A study on populations of South Asian and European backgrounds showed that ACs were associated with decreased risk of T2DM (40). Okuda et al. found no difference in plasma AC levels between patients with common diabetes and patients with non-insulin-dependent diabetes (41). Inconsistent study showed that DR patients had higher levels of AC than diabetic controls and patients with value-added retinopathy (PDR) had higher plasma carnitine levels than patients with non-proliferative retinopathy (NPDR) (42). The diabetes duration and the distribution and characteristics of HbA1c were significantly different from those in this study. In addition, the sample size of our study is much larger than that of the above study. The population selected in this study was a special analysis between people with T2DM and DR, which might cause our results to be different from previous findings. The results of the logical regression model suggested that our findings were meaningful. Therefore, we speculate that the metabolic level of ACs is different between different disease states and healthy people. A prospective study is necessary to further characterize the relationship between ACs and DR.

Our findings have important clinical and public health implications: (1) these ACs may be candidate markers for predicting the risk of DR in T2DM patients due to the increase of AUC by adding C2, C14DC, C18:1OH, and C18:1 into the model. Although these are potential predictors of DR, due to the limitations of cross-sectional studies, caution should be taken when concluding the relationship between ACs and DR; (2) fundus examination is the basis for the diagnosis and staging of DR, and our results may find new biomarkers for the early diagnosis of DR; (3) as a common complication that can seriously affect the quality of life of T2DM patients, it is important to understand the metabolic mechanism of DR before the occurrence of organic lesions. Our study provides new insight into the pathway from T2DM metabolic disorder to DR; (4) Our study comprehensively detected the levels of more than 20 kinds of plasma AC and found that the level of AC decreased generally in patients with DR, suggesting that the treatment or prevention of DR can be achieved by supplementing carnitine metabolites, which provides a new idea for the study of DR treatment.

### Limitations

Our study also has the following shortcomings. First, this study was based on a cross-sectional study of inpatients and cannot infer the causal relationship between AC accumulation and DR. Second, the diet of the subject was not collected in this study. The homeostasis of carnitine in the human body was maintained by dietary absorption, moderate synthesis rate, and effective kidney reabsorption (43). As an intermediate of FA metabolism, diet also affects the concentration of ACs in blood and urine (44). However, we adjusted the potential confounding effects of other demographic and clinical factors, which may have partially eliminated the confounding effects of eating habits. Third, because HbA1c, HDL-C and LDL-C, and TGs have missing values, the random forest method was used for interpolation, and the change of OOB error rate was very small, indicating that the interpolation results were relatively stable, and the influence on the research results was small because AC value was not missing. Fourth, the degree of disease progression of DR patients was not subdivided. Fifth, the interaction of other macrovascular and microvascular complications was not excluded.

## Conclusions

In summary, our study found that some short- and long-chain ACs were negatively correlated with the risk of DR in T2DM patients. In the future, we need to conduct larger multicenter prospective studies to further verify our findings. In addition, it is worth paying attention to whether C2, C14DC, C18:1OH, and C18:1 can be used as predictors of DR. Next, we will further study the effects of these ACs on DR and their ability to predict the risk of DR, to find biomarkers that change before organic retinopathy occurs in patients with diabetes.

## Data Availability Statement

The raw data supporting the conclusions of this article will be made available by the authors, without undue reservation.

## Author Contributions

Z-ZF, X-QG, and W-YW partly contributed to the conception and design of the work. W-YW, XinL, and XuL were responsible for organizing electronic medical records. W-YW and X-QG contributed to the statistical analysis and analyzed the data. W-YW wrote the manuscript. All authors contributed to the article and approved the submitted version.

## Funding

This work was supported by the National Key Research and Development Program of China (2021YFA1301200, 2019YFA0802302, 2019YFA0802300), Open Fund of Hubei Province Key Laboratory of Persistent Toxic Substances (PTS) Environmental Health Hazards (PTS2020-05), and the Open Fund of Tianjin Central Hospital of Gynecology Obstetrics/Tianjin Key Laboratory of Human Development and Reproductive Regulation (2020XHY01).

## Conflict of Interest

The authors declare that the research was conducted in the absence of any commercial or financial relationships that could be construed as a potential conflict of interest.

## Publisher’s Note

All claims expressed in this article are solely those of the authors and do not necessarily represent those of their affiliated organizations, or those of the publisher, the editors and the reviewers. Any product that may be evaluated in this article, or claim that may be made by its manufacturer, is not guaranteed or endorsed by the publisher.
