# Supplementary material for: Relationship Between Acylcarnitine and the Risk of Retinopathy in Type 2 Diabetes Mellitus
Source: Front Endocrinol (Lausanne). 2022 Mar 15;13:834205. doi: 10.3389/fendo.2022.834205 (PMC8964487; doi:10.3389/fendo.2022.834205)
Supplement: Supplementary file 1 [file DataSheet_1.docx]

Supplementary Material

Supplementary Table 1. OR of all Acylcarnitine for DR risk in T2DM (Multivariable model 1^a^)

| **Alyccarnitine** | **OR** | **95%CI** | **P value** |
| --- | --- | --- | --- |
| C18:1OH | 0.50 | 0.36 to 0.69 | 0.001 |
| C2 | 0.58 | 0.43 to 0.8 | 0.011 |
| C16 | 0.64 | 0.49 to 0.85 | 0.016 |
| C14DC | 0.66 | 0.51 to 0.86 | 0.016 |
| C18:1 | 0.63 | 0.46 to 0.85 | 0.016 |
| C5 | 0.71 | 0.54 to 0.95 | 0.11 |
| C18 | 0.75 | 0.58 to 0.97 | 0.119 |
| C14:1 | 0.71 | 0.53 to 0.97 | 0.119 |
| C3 | 0.72 | 0.53 to 0.97 | 0.119 |
| C18OH | 0.76 | 0.59 to 0.99 | 0.127 |
| C6 | 0.77 | 0.58 to 1.01 | 0.182 |
| C20 | 0.76 | 0.57 to 1.03 | 0.182 |
| C16OH | 0.79 | 0.61 to 1.03 | 0.182 |
| C14:2 | 0.78 | 0.6 to 1.03 | 0.182 |
| C16:1OH | 0.79 | 0.61 to 1.03 | 0.182 |
| C6DC | 0.56 | 0.28 to 1.12 | 0.195 |
| C22 | 0.81 | 0.63 to 1.04 | 0.195 |
| C14OH | 0.81 | 0.62 to 1.05 | 0.195 |
| C5DC | 0.82 | 0.63 to 1.07 | 0.261 |
| C4 | 0.86 | 0.66 to 1.12 | 0.418 |
| C5:1 | 0.87 | 0.67 to 1.12 | 0.423 |
| C14 | 0.88 | 0.68 to 1.13 | 0.481 |
| C4DC | 0.88 | 0.68 to 1.14 | 0.485 |
| C24 | 1.12 | 0.86 to 1.45 | 0.545 |
| C5OH | 1.12 | 0.85 to 1.47 | 0.545 |
| C18:2 | 0.90 | 0.7 to 1.16 | 0.545 |
| C3DC | 1.08 | 0.82 to 1.43 | 0.675 |
| C10:1 | 0.93 | 0.71 to 1.21 | 0.675 |
| C26 | 1.06 | 0.82 to 1.38 | 0.733 |
| C10:2 | 0.95 | 0.73 to 1.25 | 0.794 |
| C12 | 0.96 | 0.74 to 1.24 | 0.794 |
| C8 | 0.97 | 0.74 to 1.26 | 0.831 |
| C10 | 0.97 | 0.74 to 1.27 | 0.834 |

*Abbreviations: T2DM, type 2 diabetes mellitus; DR, diabetic retinopathy; OR, odds ratio; CI, confidence interval; C2, acetylcarnitine; C3, propionylcarnitine; C3DC, propionylcarnitine; C4, butyrylcarnitine; C4DC, succinylcarnitine; C5, isovalerylcarnitine; C5OH, 3-hydroxyisovalerylcarnitine; C5DC, glutarylcarnitine; C5:1, tiglylcarnitine; C6, hexanoylcarnitine; C6DC, adipylcarnitine; C8, octanoylcarnitine; C10, decanoylcarnitine; C10:1, decanoylcarnitine; C10:2, sebacoylcarnitine; C12, lauroylcarnitine; C14, myristoylcarnitine; C14OH, 3-hydroxyl-tetradecanoylcarnitine; C14DC, tetradecanoyldiacylcarnitine; C14:1, tetradecenoylcarnitine; C14:2, tetradecadienylcarnitine; C16, palmitoylcarnitine; C16OH, 3-hydroxypalmitoylcarnitine; C16:1OH, 3-hydroxypalmitoleylcarnitine; C18, octadecanoylcarnitine; C18OH, 3-hydroxy-octadecoylcarnitine; C18:1, octacarbonylcarnitine; C18:1OH, 3-hydroxy-octadecylcarnitine; C18:2, octadecadienylcarnitine; C20, arachidic carnitine; C22, behenic carnitine; C24, tetracosanoic carnitine; C26, hexacosanoic carnitine.*

*^a^Multivariable model 1 was adjusted for age, sex, body mass index (<18.5 kg/m2, 18.5 kg/m2-24.0 kg/m2, 24.0 kg/m2-28.0 kg/m2 and >28.0 kg/m2), SBP, DBP, and diabetes duration on the basis of univariable model.*

Supplementary Table 2. OR of all Acylcarnitine for DR risk in T2DM (Multivariable model 2^a^)

| **Alyccarnitine** | **OR** | **95%CI** | **P value** |
| --- | --- | --- | --- |
| C18:1OH | 0.51 | 0.36 to 0.71 | 0.003 |
| C2 | 0.55 | 0.39 to 0.76 | 0.006 |
| C14DC | 0.64 | 0.49 to 0.84 | 0.010 |
| C16 | 0.63 | 0.47 to 0.83 | 0.010 |
| C18:1 | 0.60 | 0.44 to 0.83 | 0.012 |
| C3 | 0.68 | 0.5 to 0.93 | 0.091 |
| C5 | 0.71 | 0.53 to 0.95 | 0.091 |
| C18 | 0.75 | 0.57 to 0.97 | 0.113 |
| C14:1 | 0.71 | 0.51 to 0.97 | 0.113 |
| C6 | 0.74 | 0.55 to 0.98 | 0.113 |
| C18OH | 0.76 | 0.58 to 0.99 | 0.129 |
| C16:1OH | 0.77 | 0.58 to 1.01 | 0.151 |
| C14:2 | 0.76 | 0.57 to 1.02 | 0.175 |
| C14OH | 0.78 | 0.59 to 1.03 | 0.188 |
| C20 | 0.77 | 0.57 to 1.04 | 0.188 |
| C22 | 0.80 | 0.62 to 1.05 | 0.204 |
| C16OH | 0.80 | 0.62 to 1.05 | 0.204 |
| C4 | 0.80 | 0.61 to 1.06 | 0.204 |
| C6DC | 0.54 | 0.25 to 1.17 | 0.204 |
| C5DC | 0.81 | 0.61 to 1.09 | 0.283 |
| C5:1 | 0.85 | 0.66 to 1.09 | 0.321 |
| C4DC | 0.87 | 0.67 to 1.13 | 0.435 |
| C18:2 | 0.89 | 0.69 to 1.15 | 0.530 |
| C14 | 0.90 | 0.69 to 1.16 | 0.568 |
| C10:1 | 0.90 | 0.69 to 1.18 | 0.568 |
| C24 | 1.11 | 0.85 to 1.45 | 0.568 |
| C5OH | 1.11 | 0.83 to 1.47 | 0.591 |
| C12 | 0.93 | 0.71 to 1.21 | 0.677 |
| C10 | 0.93 | 0.71 to 1.23 | 0.694 |
| C8 | 0.94 | 0.72 to 1.23 | 0.694 |
| C3DC | 1.07 | 0.8 to 1.42 | 0.694 |
| C26 | 1.05 | 0.8 to 1.36 | 0.754 |
| C10:2 | 0.95 | 0.72 to 1.27 | 0.754 |

*Abbreviations: T2DM, type 2 diabetes mellitus; DR, diabetic retinopathy; OR, odds ratio; CI, confidence interval; C2, acetylcarnitine; C3, propionylcarnitine; C3DC, propionylcarnitine; C4, butyrylcarnitine; C4DC, succinylcarnitine; C5, isovalerylcarnitine; C5OH, 3-hydroxyisovalerylcarnitine; C5DC, glutarylcarnitine; C5:1, tiglylcarnitine; C6, hexanoylcarnitine; C6DC, adipylcarnitine; C8, octanoylcarnitine; C10, decanoylcarnitine; C10:1, decanoylcarnitine; C10:2, sebacoylcarnitine; C12, lauroylcarnitine; C14, myristoylcarnitine; C14OH, 3-hydroxyl-tetradecanoylcarnitine; C14DC, tetradecanoyldiacylcarnitine; C14:1, tetradecenoylcarnitine; C14:2, tetradecadienylcarnitine; C16, palmitoylcarnitine; C16OH, 3-hydroxypalmitoylcarnitine; C16:1OH, 3-hydroxypalmitoleylcarnitine; C18, octadecanoylcarnitine; C18OH, 3-hydroxy-octadecoylcarnitine; C18:1, octacarbonylcarnitine; C18:1OH, 3-hydroxy-octadecylcarnitine; C18:2, octadecadienylcarnitine; C20, arachidic carnitine; C22, behenic carnitine; C24, tetracosanoic carnitine; C26, hexacosanoic carnitine.*

*^a^Multivariable model 2 was adjusted for variables in multivariable model 1 plus glycated hemoglobin (<7%, ≥7%), triglyceride (<1.7mmol/L, ≥1.7mmol/L), high-density lipoprotein cholesterol (<1.0mmol/L in male or <1.3mmol/L in female, ≥1.0mmol/L in male or ≥1.3mmol/L in female), and low-density lipoprotein cholesterol (<2.6mmol/L, ≥2.6 mmol/L).*
